# Perceptions, facilitators and barriers to the implementation of interpersonal group therapy to treat depression among people living with HIV in Senegal: a qualitative study

**DOI:** 10.3389/fpubh.2024.1295181

**Published:** 2024-01-24

**Authors:** Charlotte Bernard, Idrissa Mané, Salaheddine Ziadeh, Judicaël Malick Tine, Abibatou Diaw, Noëlle Benzekri, Ibrahima Ndiaye, Oumar Samba, Hélène Font, Thierry Bottai, Laurent Jacquesy, Helen Verdeli, Ndeye Fatou Ngom, François Dabis, Moussa Seydi, Nathalie de Rekeneire

**Affiliations:** ^1^National Institute for Health and Medical Research (INSERM) UMR 1219, Research Institute for Sustainable Development (IRD) EMR 271, Bordeaux Population Health Centre, University of Bordeaux, Bordeaux, France; ^2^CNRS, Université de Pau et des Pays de l’Adour, Pau, France; ^3^Université Libanaise, Faculté de Santé Publique, Sidon, Lebanon; ^4^Global Mental Health Lab, Teachers College, Columbia University, New York, NY, United States; ^5^Service des maladies infectieuses et tropicales, CHNU de Fann, Dakar, Senegal; ^6^Centre de Traitement ambulatoire, CHNU de Fann, Dakar, Senegal; ^7^Department of Medicine-Infectious Diseases, University of Washington, Seattle, WA, United States; ^8^Service de psychiatrie, CHNU de Fann, Dakar, Senegal; ^9^Pôle de psychiatrie, CH de Martigues, Martigues, France; ^10^Psychiatre indépendant, président de CREATIP, Annecy, France; ^11^Institut Pasteur du Cambodge, Phnom Penh, Cambodia

**Keywords:** HIV, depression, sub-Saharan Africa, group interpersonal therapy, mental health

## Abstract

**Background:**

Depression is highly prevalent in people living with HIV (PLWH) but remains under treated in Sub-Saharan Africa. In this context, we conducted the first study of Group Interpersonal Therapy (IPT) to treat depression in PLWH in Senegal. We assessed the perceptions and experiences of patients and group facilitators, as well as barriers to implementation.

**Methods:**

This study was conducted at the Fann National University Hospital Center in Dakar, the urban capital of Senegal. Qualitative data were collected during the implementation phase (February to June 2020 and then from January to February 2021), with a 6-month pause due to the COVID-19 pandemic. Twenty-five patients and three group facilitators were individually interviewed by a socio-anthropologist. Qualitative data were analyzed thematically.

**Results:**

Group IPT was perceived as successful and beneficial by patients and facilitators. Patients reported positive experiences with group IPT and sustained outcomes. Beyond improving depressive symptoms, patients reported improvements in their social and professional lives, and the development of skills to prevent relapse. Group facilitators noted the benefits of therapy for their patients and for their professional skills, reporting greater clinical competence and improved supportive skills. Challenges to intervention implementation included confidentiality and patient privacy concerns, healthcare accessibility issues, and time demands.

**Conclusion:**

In this first qualitative study of group IPT for depression in PLWH in Senegal, participants described both positive experiences with the intervention and challenges to its implementation. Future studies, conducted in suburban and rural communities outside of Dakar, would further inform the implementation of IPT in Senegal.

## 1 Introduction

Depression can occur at any stage of life and is one of the most frequent mental health disorders, with about 280 million people worldwide living with a depressive disorder as of 2019 (1). To treat depression, the World Health Organization (WHO) recommends psychotherapy as a first line treatment.

Interpersonal therapy (IPT) was first developed (2) as an individual psychotherapy for the treatment of depression. It posits that interpersonal problems—such as disagreement or conflict, life changes (negative or positive), grief, loneliness or social isolation—may trigger depressive symptoms (3–5) and that patients can overcome their depression by building skills to better manage these problem areas. This is typically achieved within a limited number of sessions as patients develop interpersonal competencies and learn strategies to successfully tackle their interpersonal problems and even avert a future relapse. Since its inception as treatment for depression, IPT has been successfully adapted to different pathologies, intervention modalities, age groups, and care sites around the world (6). Its effectiveness in the management of depression has been shown in systematic reviews (7, 8). As a group intervention, it was first applied in Sub-Saharan Africa (SSA) in Uganda with encouraging outcomes (9–11).

In low and middle-income countries (LMIC), there is an enormous gap in mental health care. This has been attributed, among other things, to the scarcity of mental health specialists (12). In SSA, depression often remains underdiagnosed and undertreated (13, 14). The scarcity of mental health resources and the high prevalence of depression present a double burden that calls for efficiency and smart use of resources. As group IPT is effective and compatible with task-shifting (15, 16), the World Health Organization (WHO) specifically recommends it as treatment for depression in LMIC (17).

Depression is the most common neuropsychiatric disorder in people living with HIV (PLWH) (13) and has high prevalence in SSA according to recent meta-analyses (13%–14%) (18, 19). Depression is associated with negative HIV outcomes (i.e., low adherence to antiretroviral therapy (ART), rapid progression to AIDS stage, slower increase in CD4 count) (14, 20, 21) and negative impacts on quality of life (13). IPT has been shown to be an effective method to treat depression in PLWH in multiple settings. In a randomized controlled trial in the United States, IPT showed higher efficacy in treating depression in PLWH, compared to medication, cognitive behavioral and supportive psychotherapy (22). Recently, group IPT was reported feasible and acceptable in PLWH in Northwest Ethiopia (23) and to significantly improve depressive symptoms in PLWH in South Africa compared to a control group (24).

Our present study is the first time where group IPT, delivered via a task-shifting approach, was implemented to treat depression in PLWH in Senegal, with high acceptability, feasibility and benefits to patients (25). To better understand the patient and provider participants’ experiences, we conducted qualitative interviews with both parties. A recent qualitative study in Northern Ethiopia reported the experience of implementing group IPT for PLWH (26) but provided no information on patients’ or facilitators’ experience with group IPT. In West Africa, where Senegal is geographically located, the published data on IPT implementation is simply nonexistent.

Using a qualitative approach, we aimed to: (1) explore patients’ perceptions and experiences with group IPT (hereafter, “IPT-participants”); (2) understand the perceptions and experiences of group facilitators (“IPT-facilitators”); and (3) identify facilitative factors (“facilitators”) and barriers to group IPT implementation.

## 2 Methods

### 2.1 Setting

The present study was conducted in Dakar, Senegal, at the Fann National University Hospital Center. Specifically, it was carried out in two hospital units: (1) the Infectious and Tropical Diseases Service and (2) the Outpatient Treatment Center. Research activities were conducted in collaboration with the psychiatry department.

### 2.2 Study design

We conducted a qualitative study to better understand patient and provider experiences with group IPT using interviews with both parties. Qualitative research has historically been used to better understand participants’ beliefs, experiences, and attitudes toward an intervention (27, 28). It gives voice to study participants (29) and can provide a deeper understanding of health related interventions (27). In the present study, the qualitative approach was used to gain critical information on aspects of group IPT that could not be captured by quantitative data alone, with the aim to enrich our understanding of the treatment and its implementation.

### 2.3 Group IPT

The intervention was implemented according to the WHO manual, which had been translated to French by our team.^1^ Group IPT was delivered over the course of eight weeks, in the form of weekly group sessions preceded by a single pre-group individual meeting, one per patient. The eight weekly sessions covered the three phases of IPT treatment: initial, middle, and termination. Group facilitators were Senegalese social workers or community health workers. They were experienced in conducting HIV counseling (pre- and post-diagnosis), group psychoeducation on HIV infection, and group discussions on adherence to antiretroviral treatment; however, they had no specialized training on diagnosis and treatment of depression.

The prospective group facilitators were trained in group IPT, which consisted of a 5-day didactic and practical training (e.g., demos and role plays) followed by supervised practice (i.e., facilitation of 2 consecutive IPT groups). Supervision was conducted weekly throughout the training phase and continued into the study phase, to ensure proper treatment implementation. Training and supervision were conducted by a group IPT expert trainer (SZ). Three group facilitators completed the training and participated in the present study.

The structure of the treatment was informed by cultural considerations. Consistent with group IPT practices elsewhere in Africa, groups were gender-specific (facilitator included) given the sensitivity of discussing intimate topics with people of the opposite sex. Each group consisted of 6 IPT-participants and one group IPT-facilitator and were conducted in Wolof and/or French, the national language of Senegal. Since the term « depression » was largely associated with « craziness » in Senegal and thus highly stigmatized, the Wolof expression “naxaru xol” (literally, “loss of appetite for doing things” in Wolof) was chosen to refer to depression. The substitute term was proposed by the study site’s Senegalese psychiatrists based on their clinical experience and was approved by the social workers, based on cultural understanding and sensitivity. In session (pre-group and subsequent group sessions), confidentiality rules were clearly explained and emphasized as was the importance of attendance. Transportation costs were reimbursed, in the form of a standardized amount given at the end of each session.

### 2.4 Recruitment of participants

This qualitative study was nested in a parent study conducted within the West Africa network of the International epidemiological Databases to Evaluate AIDS (IeDEA) of the US National Institutes of Health^2^ and that evaluated the acceptability and feasibility of group IPT among PLWH in Dakar using a quantitative approach (25). For the qualitative component of the study, IPT-participants had to have completed their group IPT therapy. When the qualitative study began, 36 PLWH had already satisfied this requirement. To include a variety of perspectives, we selected 25 IPT-participants to participate in the present study using maximum variation sampling (30) with regard to age, gender, marital status, occupation, and ethnicity. Selection also depended on participant availability. We additionally interviewed all three IPT-facilitators (the two social workers and the community health worker who had facilitated the IPT groups) to understand their experience with treatment implementation.

### 2.5 Data collection

A Senegalese socio-anthropologist (IM) conducted semi-structured individual interviews with participants during two time periods (February–June, 2020; January–February, 2021), split by the pandemic-necessitated lockdown. Interviews with IPT-participants were conducted in French, Wolof or Pulaar (languages spoken fluently by the interviewer), according to the preference of each interviewee; they took place 2 weeks to 6 months following the end of their group treatment in private onsite offices associated with the project. The interview guide, used with IPT-participants, centered on: (1) perception and experience of group IPT (before and during therapy); (2) impact of the intervention on depression, participants’ lives, HIV infection perception and engagement in HIV care. A second interview guide was developed to document the perceptions and experiences of IPT-facilitators leading groups and implementing IPT. The interview guide was developed (in French) by IM, with input from the project manager (CB) in Senegal. The interviews, which were audio-recorded then transcribed by the interviewer (IM), ranged in length from 45 to 90 min. Those conducted in French were transcribed *verbatim*, whereas those in Wolof or Pulaar were directly translated and transcribed in French. Following transcription, the recordings were deleted and interview transcripts were anonymized. Excerpts included in this publication were translated from the French transcripts into English. The present study followed the Standards for Reporting Qualitative Research guidelines (31, 32).

### 2.6 Analyses

Interviews were analyzed using a classical thematic analysis (30, 33). Themes were defined using an inductive approach, based on observations made by the research team during the group IPT training phase and also based on recurrent emerging themes discussed by the interviewees. A codebook was developed based on these themes. Then, all transcripts were read and color-coded using Dedoose version *9.0.86* (cloud application for managing, analyzing, and presenting qualitative and mixed method research data *(2023)*. Los Angeles, CA: SocioCultural Research Consultants, LLC, www.dedoose.com). Recurring sub-themes were also identified, color-coded and summarized. To represent each theme or sub-theme, quotations were selected and reported as the study results. Strategies to enhance rigor included focused discussions of emerging themes with the treatment team, along with cultural contextualization (i.e., discussion of themes with attention to cultural context). These strategies helped the research team to better understand patient perceptions and experiences as well as how some of these were influenced by culture, such as local practices (e.g., “parenté à plaisanteries,” an ancestral social practice) and beliefs (e.g., faith). The aim was to elucidate pertinent data, corroborate findings, and ultimately ensure a better informed interpretation. For IPT-participants, data analysis continued until theme saturation was reached. However, all 3 IPT-facilitators were interviewed to obtain their perception and experience. Participant characteristics were described using numbers and proportions for categorical variables, and median and interquartile ranges for continuous variables.

### 2.7 Ethical approval

Ethical approval was obtained from the national ethics committee of Senegal: Conseil National d’Ethique de la Recherche en Santé (CNERS) (Protocol number: SEN18/75; approved number: 0006/MSAS/DPRS/). The study’s physicians involved in the project informed the participants of the objectives of the study and invited them to read the information notice. For those who could not read, the physicians read and explained the notice. All participants gave their written consent to participate, prior to their inclusion in the study.

## 3 Results

### 3.1 Demographic characteristics of study participants

Twenty-five PLWH were included as IPT-participants in this qualitative study. The median age was 46 years (interquartile range: 38–50 years of age). Eleven IPT-participants were women (44%); fourteen were married (56%), and fourteen were unemployed (56%). The “unemployed” category included 1 housewife and 4 university students. Most employed IPT-participants were engaged in activities with limited or irregular income. The entire team of IPT-facilitators consisted of two female social workers (aged 38 and 53 years, respectively) and a male community health worker (aged 45 years).

Key findings from the qualitative analysis are reported in Table 1.

**Table 1:** Topics, themes, and subthemes.

**Topic 1: IPT-participants’ perceptions and experiences with group IPT**
**Mixed feelings regarding participation in group IPT (i.e., before starting or in its early phase)**
Positive feelings towards group IPT
Doubts about the effectiveness of group IPT
**Recognition of group IPT as effective treatment for depression**
**Centrality of group cohesion**
**Improved knowledge about depression**
Symptom recognition
Corrected misconceptions
**Positive impacts on daily life**
Improvement in social life (connectedness and interpersonal relatedness)
Improvement in professional life
**HIV-specific changes**
Increased engagement in HIV care
More accurate perception of HIV infection
**Topic 2: IPT-facilitators’ perceptions and experiences with group IPT**
**Perceptions of group IPT training**
**Perception of the progress of group IPT**
**Challenges in the new role of facilitator**
Emotional commitment to patients
Time investment and management
**Positive impacts of group IPT on facilitators’ professional skills**
More accurate understanding of depression
Increased awareness of the barriers and challenges faced by PLWH
Improved assessment skills (distinguishing between social and mental health needs)
**Topic 3: Facilitators and barriers to group IPT implementation**
**A call for capacity building and IPT dissemination**
**Sociocultural facilitators**
Ancestral social practice (i.e., “parenté à plaisanterie”)
Patient religion and faith
**Centrality of confidentiality and trust**
Patient fear of disclosure to other group members
Threat to patient privacy (in the context of treatment location’s association with HIV)
**Challenges to healthcare access**
Identification of depressed patients, systemic challenges
Access to care for key populations is fraught with difficulties (e.g., stigma)
Transportation costs
**Time burden for IPT-participants**
**Limited cultural applicability to the Senegalese context of some case examples in the IPT manual**

### 3.2 TOPIC 1: IPT-participants’ perceptions and experience with group IPT

#### 3.2.1 Mixed feelings regarding participation in group IPT (i.e., before starting or in its early phase)

##### 3.2.1.1 Positive feelings towards group IPT

For the majority of participants, the invitation to join an IPT group was easily accepted. There were positive feelings, such as relief, confidence, hope of being helped, and the possibility of beneficial interactions with peers.

*“I thought it would make me feel better. That’s why I came to the group. I felt confident that group therapy could help me. So I welcomed the proposal [to join] with hope.”* [IPT-Participant 25].

*“I welcomed it because bringing together people who have the same problem, the same stress, allows them to know they are not the only ones in the world to experience this. Thus, they can better approach life.”* [IPT-Participant 13]

Some participants appreciated that medical staff cared about their mental health and not just their physical health. Some were relieved that their condition of “being unwell” was identified by the staff.

*“It is reassuring to know that doctors are also concerned about our mental health. They could have limited themselves to treating the disease [HIV infection].”* [IPT-Participant 14].

##### 3.2.1.2 Doubts about the effectiveness of group IPT

Some participants expressed doubt about the effectiveness of group IPT (i.e., whether it could work, especially in a group setting, and how talking about problems could help).

*“Honestly, I thought to myself that mere words could not take away my depression.”* [IPT-Participant 9].

There was also some reluctance about sharing personal problems with strangers, with a notable ambiguity about how sharing in group could help.

*“I thought to myself, “How can it help me to talk to people I do not know, and how can I share my problems when they do not even know me?”* [IPT-Participant 12].

To some participants, it felt unnatural or strange, with a reluctance to self-disclose. However, the hesitation was overcome with time, as sharing in group became associated with feeling better.

*“The first few moments were a little strange: having to sit around a table with strangers and expose my problems. That’s it! [laughs] As a responsible person and a father, it was not easy to expose my problems to other people… But as time went by, I got the courage and the desire to speak. It made me feel better.”* [IPT-Participant 20].

#### 3.2.2 Recognition of group IPT as effective treatment for depression

Initial doubts quickly dissipated and gave way to hope about therapy and the desire to share stories with others. Some patients reported that, through group participation, they realized how sharing their struggles in an open and interactive manner could help them overcome their problems and get better.

*“I saw that it was going well and that it was an open question-and-answer discussion with the other group members. I realized that therapy could help us without the need to take medication. Within the first few moments of therapy, I was already feeling better.”* [IPT-Participant 2].

Group IPT was seen as a way of opening up to others in a safe and trusting environment. It allowed some IPT-participants to put their situation in perspective, draw solutions from the experience of others, and to socialize again through interpersonal interaction.

*“Meeting people who have problems just as complex as mine or even more difficult than mine allowed me to put my pain in perspective, to accept it and fight to change my condition. So therapy has only advantages.”* [IPT-Participant 6].

IPT-participants reported helping each other, with a growing sense of unity and closeness in the group, and the desire to come and share their struggles in order to move forward. The sense of mutual support and belonging was in itself therapeutic.

*“In the group, after the first few days, I felt confident and supported. I was no longer facing my problems alone. I had a whole team with me.”* [IPT-Participant 18].

#### 3.2.3 Centrality of group cohesion

The majority of IPT-participants spoke positively of the cohesiveness that existed among group members in session and out of session. The desire to return to subsequent sessions was also mentioned several times. Mutual trust and support developed in group. IPT-participants maintained contact beyond group sessions, by phone and social media (e.g., *WhatsApp* group), mutual visits, and lunch gatherings. Some participants did not want the group to end.

*“We did not even want the sessions to end because we had become a family. We knew each other well; we called each other by phone. We had suggested that meetings continue for a while, but it was not possible for therapy to continue beyond a certain number of sessions.”* [IPT-Participant 1].

#### 3.2.4 Improved knowledge about depression

##### 3.2.4.1 Symptom recognition

In addition to improvements in depressive symptoms and daily living, IPT-participants felt confident that they could recognize depressive symptoms in future episodes should one occur. What they had learned in the group, they said, would help them cope with an eventual relapse. Moreover, they could identify depressive symptoms in others and would help them.

*“Honestly, IPT has a real purpose, a real importance. I think I can prevent depression in myself, identify it in others and try to combat it with the approach I learned in group sessions.”* [IPT-Participant 19].

##### 3.2.4.2 Corrected misconceptions

Group IPT helped IPT-participants correct some of the misconceptions about depression, often associated in Senegal with the West and individualistic cultures. They learned that depression was a separate illness from HIV infection, could affect anyone, and that it required treatment. IPT-participants shared that depression could be treated by talking, sharing one’s experience, and opening up to others. They differentiated depression from “madness.” Those who had associated depression with death (or had wanted to end their lives) overcame their morbid and suicidal thoughts.

*“My view of depression has really changed because of our meetings. I now know that it can be treated with words, without medication. I also know that it is not a symptom of the disease [HIV infection] we have.”* [IPT-Participant 3].

*“Thanks to therapy, I now know that depression is a normal thing that can happen to anyone.”* [IPT-Participant 15].

*“I know from therapy that what was happening to me had nothing to do with madness.”* [IPT-Participant 25].

#### 3.2.5 Positive impacts on daily life

##### 3.2.5.1 Improvement in social life (connectedness and interpersonal relatedness)

The majority of IPT-participants reported that their lives had improved significantly in the social and professional domains following group IPT. They reported being closer to their families and/or friends, being more likely to participate in family life and social activities, and better at handling adversity and interpersonal difficulties. Some IPT-participants reported reconciling with people with whom they had disagreements.

*“Since I came out of that group therapy, I have become closer with family and friends. […] Sometimes I go out for some fresh air with relatives or friends. Mashallah. This change has brought back friends who had distanced themselves from me because of my self-isolation.”* [IPT-Participant 22].

##### 3.2.5.2 Improvement in professional life

Group IPT also enabled them to return to work, start a new business, or better plan the development of their current business. For two IPT-participants who were students, IPT enabled them to return to school.

*“The therapy changed my life and restored my life plans. I regained hope and went back to school.”* [IPT-Participant 12].

#### 3.2.6 HIV-specific changes

##### 3.2.6.1 Increased engagement in HIV care

IPT-participants also reported a greater motivation to adhere to medication and keep medical appointments. This included those already engaged in their healthcare who, with group IPT, became even more committed to their ART.

*“With the therapy and guidance of the group and medical staff, I resumed the treatment properly and my viral load at my last check-up was truly undetectable. I was very happy and so was the doctor.”* [IPT-Participant 15].

##### 3.2.6.2 More accurate perception of HIV infection

Some misconceptions about HIV infection were apparently rectified through group IPT, with a number of IPT-participants reporting a less fatalistic view of the infection—describing it as a chronic illness with which you can live and have a future.

*“I really carried the disease like a burden. But, since the group meetings, I know that it is not worth doing so. Because it is a disease like any other, just like diabetes.”* [IPT-Participant 8].

The exchange of experiences between the young and the older adult in some groups also helped to put things in perspective (e.g., normalization; reassurance). It especially helped patients who had difficulty accepting their HIV disease.

“*In the beginning, I thought that the disease would take me very soon […] But since I’ve been in the group and older women said that they had been on their treatment for several years without having any problems, my perception has changed.”* [IPT-Participant 10].

### 3.3 TOPIC 2: IPT-facilitators’ perceptions and experiences with group IPT

#### 3.3.1 Perceptions of group IPT training

IPT-Facilitators indicated that the 5-day IPT program of didactic and practical training was “excellent”; nevertheless, they would have appreciated more face-to-face time with the trainer to better master the various techniques and the course of therapy.

*“In my training, the difficulty I can highlight is the short duration of the course. Since it was my first experience, it would have been good to do the training over and over again. However, we later had skill building [through supervised practice with patient groups].”* [IPT-Facilitator 3].

#### 3.3.2 Perception of the progress of group IPT

IPT-Facilitators reported a warm ambiance, mutual support and cohesion in groups they ran. They also endorsed the utility of being in a group, to stimulate exchanges and find solutions to the problems faced by patients. Subsequently, they were pleased to see their former patients do better and be grateful during follow-up visits. They underscored the group’s ability to help their patients.

*“It’s good for the patients and for us because there’s nothing better than seeing patients get better and keep their medical appointments and take their medications. IPT-G helps me, as a facilitator, to find solutions for patients. It’s true that the ideas do not come from me in the IPT-G sessions but rather from the participants themselves. I think that if I had to work individually with each patient, I might not be able to find the solutions as easily as in group.”* [IPT-Facilitator 2].

A number of strategies and techniques learned in the 5-day training (and manual) helped the IPT-facilitators better manage time and tension in group. The group modality, they stated, was more advantageous in terms of patient management and time use, compared to individual therapy.

*“It is really a breath of fresh air because if we had to follow each depressed patient individually, we would not have had the same results and it would have taken a lot of time.”* [IPT-Facilitator 2].

#### 3.3.3 Challenges in the new role of IPT-facilitator

##### 3.3.3.1 Emotional commitment to patients

One facilitator indicated that it was sometimes more difficult to listen to participants’ stories than to look for solutions, inasmuch as some could be emotionally intense at times. Another spoke about the difficulties with a participant who went through several sessions without showing any sign of improvement and how challenging that was.

*“I feel that patient problems are kind of a burden. That’s the difficulty. The hard part is receiving the patient’s problems, not solving them.”* [IPT-Facilitator 1].

*“Sometimes, there are problems so serious that no matter how much advice is given, no matter how much support is given by group members, when the patient gets home and tries to move forward, he really does not move enough. And, even if there is improvement, it is very, very minimal. Sometimes there is none. For me, it is complicated.”* [IPT-Facilitator 2].

##### 3.3.3.2 Time investment and management

IPT-facilitators said time management could be an issue at times, either because IPT-participants arrived late to session or because, with six members in the group, they had to prioritize issues in order to hear from all IPT-participants without exceeding the session time limit, a common therapist developmental issue.

*“The disadvantages are that you cannot deal with 6 problems that may come up simultaneously in one day, in one session. The facilitator is forced to take a few issues, to focus.”* [IPT-Facilitator 1].

Two IPT-facilitators shared that group IPT took time and required an investment on their part, especially to manage the time of IPT sessions, to complete patients’ records and to participate in supervision sessions.

*“The facilitator has to be really invested in order to be successful in conducting the therapy or it will not work. He/she must be able to manage time, expectations, and everything beyond the conduct of the therapy itself.”* [IPT-Facilitator 1].

#### 3.3.4 Positive impacts of group IPT on IPT-facilitators’ professional skills

##### 3.3.4.1 More accurate understanding of depression

Group IPT seems to have changed IPT-facilitators’ perceptions of depression and its treatment. Group IPT training helped them recognize the symptoms and causes of depression, in particular the fact that depression is not linked to madness. They also realized the importance of talking to their patients and the possibility of helping them through psychotherapy.

*“I did not know before what depression really was. I also did not know that by talking and providing support, you could help a person get out of depression without necessarily using medication and all that. […] Also, the training allowed me to differentiate between depression and madness, in terms of mental health.”* [IPT-Facilitator 1].

##### 3.3.4.2 Increased awareness of the barriers and challenges faced by PLWH

IPT-facilitators stated that they gained insight into the challenges faced by PLWH and why some IPT-participants were still having difficulty adhering to their HIV treatment. They identified a non-judgmental stance and a better understanding of PLWH, including their histories and life realities, as necessary to supporting and helping them.

*“I used to be somewhat intolerant of patient non-adherence to treatment. IPT completely changed this by allowing me to better understand the relationship between HIV infection, mental health, and the* var*ious problems that HIV patients faced.”* [IPT-Facilitator 2].

*“Since the training, I have come to understand the reality of living with HIV, including the mental health implications and the kind of vicious cycle that exists between HIV infection and subsequent life problems that [in turn] affect the infection itself, especially when patients are non-compliant with HIV treatment.”* [IPT-Facilitator 1].

##### 3.3.4.3 Improved assessment skills (distinguishing between social and mental health needs)

The impact of group IPT on facilitators’ professional competence was palpable, both in terms of practice and relationship with patients, through skill acquisition and development. Whereas previously they were limited to counseling and education about HIV and its management, now they can intervene therapeutically with a patient. They also learned to better differentiate the social from the psychological needs of their patients.

*“Now that I have done the IPT-G training, I know when therapeutic education is needed and when interpersonal therapy can solve the problem.”* [IPT-Facilitator 1].

*“I had been in contact with depressed people with HIV, but never dealt with depression. Our work with these patients was limited to social assistance. […] As a result of my IPT-G training, however, now it is different. Even within the scope of my work as social worker, I reach my goals quicker [with IPT].”* [IPT-Facilitator 3].

### 3.4 Topic 3: Facilitators and barriers to group IPT implementation

#### 3.4.1 A call for capacity building and IPT dissemination

IPT-facilitators valued the effectiveness of group IPT and called for capacity building and dissemination across the country.

*“It is therefore necessary to democratize and facilitate the use of IPT-G. If we can decentralize IPT-G, it would be a very good thing, but we must also train people*.” [IPT-Facilitator 2].

*“All social workers, even those who do not work with PLWH, need to be trained [in IPT] to bridge the gap between what we need to learn and what is actually taught in social work programs*.” [IPT-Facilitator 3].

A number of IPT-participants thanked the treatment team for providing them with therapy and asked whether group IPT could be disseminated to benefit other patients.

*“I know that many people suffer from depression but do not get help. That’s why I’m asking you to spread this very useful therapy.”* [IPT-Participant 18].

#### 3.4.2 Sociocultural facilitators

##### 3.4.2.1 Ancestral social practice (i.e., “parenté à plaisanterie”)

IPT-Facilitators spontaneously resorted to an ancestral social practice, called the “parenté à plaisanterie” (34, 35) to animate groups. The term refers to a culturally-sanctioned mode of interaction between two people of different ethnicities, where one is allowed to tease the other without the latter taking offense.

“*This [mode of interaction] allows me to say things to patients without alienating them, and to build trust and group cohesion. Also, it allows me to take a patient out of his comfort zone and put what is happening with him in perspective*.” [IPT-Facilitator 1].

This practice appears to have occurred naturally in various groups as a familiar method to diffuse tension and build cohesion, thus deepening mutual trust.

*“I also used it in the beginning, to build trust between group members as well as between them and me.”* [IPT-Facilitator 2].

##### 3.4.2.2 Patient religion and faith

IPT-Facilitators also drew on faith to raise morale, lower risk of self-harm, engage patients in the process of recovery, and help them move forward.

*“I tend to think that faith helps the therapy because we are believers and belief plays an important role in the mental constitution of the Senegalese*.” [IPT-Facilitator 1].

*“I think that faith in God plays a positive role especially at the individual level, since it discourages suicidal tendencies. Yes, it prevents suicide and it moves people towards acceptance*.” [IPT-Facilitator 2].

#### 3.4.3 Centrality of confidentiality and trust

##### 3.4.3.1 Patient fear of disclosure to other group members

Respect for confidentiality rules in group along with IPT-participants’ trust in treatment providers were critical to IPT success. This assertion was endorsed by IPT-participants and IPT-facilitators alike. Some IPT-participants initially expressed a reluctance (and sometimes fear) to participate, often in the form of a confidentiality breach or running into someone they knew at the hospital or in group.

*“I was a little stressed imagining that I might be in the same group as someone I know from the neighborhood. Also, I was wondering whether these people in the group could be as discrete as I would be.”* [IPT-Participant 19].

*“As to challenges, they are mostly felt in the pre-group phase, particularly the fact that patients are afraid of having to talk about their HIV status in group…, their fear of a confidentiality breach. I sensed this fear in my patients.”* [IPT-Facilitator 3].

Another challenge was weekly group attendance. Inasmuch as IPT groups did not necessarily take place on the same day as other healthcare appointments, suspicion could arise in a participant’s entourage about their extra hospital visits.

*“The only downside is that it requires you to come to the hospital every week, outside of other medical appointments. After a while, we have to hide from people that we are going to the hospital*.” [IPT-Participant 5].

##### 3.4.3.2 Threat to patient privacy (in the context of treatment location’s association with HIV)

The setting for IPT treatment (i.e., Fann hospital) was associated with safety and wellness in the mind of patients inasmuch as it was also the place of their medical care. Trust in, and familiarity with, the hospital staff contributed to the psychological sense of safety. In this context, IPT-participants did not fear that their HIV status or confidential records would be disclosed, a reason for which the majority considered the hospital to be the safest place for group psychotherapy.

*“I prefer to have the sessions here because I do my treatments here and I know all the staff at the center*.” [IPT-Participant 7].

In figuring out the optimal place for running IPT groups, some IPT-participants deferred the decision to the treatment team while others reasoned that providing therapy outside the hospital would increase patient access to IPT. Although a hospital provides broader anonymity in terms of *who* is there getting *what*, given the multitude of services it offers, an implicit HIV status disclosure cannot be entirely averted in view of frequent patient visits to a place associated with HIV care.

*“I leave the choice up to the medical team, since I have a complete confidence in the decisions made here. If you think it’s better to meet in one place than another, then you have the right reasons.”* [IPT-Participant 19].

#### 3.4.4 Challenges to healthcare access

##### 3.4.4.1 Identification of depressed patients, systemic challenges

Patient access to healthcare services is contingent on several factors, including service seeking behavior and successful identification of patients. Whereas the latter is mainly procedural (e.g., systematic screening), the former is largely sociocultural. Even when patients present for services, they still can easily discontinue them. Since depression is still stigmatized in Senegal, IPT-facilitators needed some extra time to screen for depression, and to de-stigmatize the illness with adequate psychoeducation so that patients could be diagnosed, join and participate in groups.

*“It’s not easy to recruit patients. Sometimes, you pick up a [potential] patient, only to lose them. If you do not take time to explain [the problem] well because you are in a hurry, you lose the person. Sometimes, all it takes is a word about depression, and the patient will tell you “no, no, doffouma” [“no, no, I’m not crazy”].”* [IPT-Facilitator 3].

##### 3.4.4.2 Access to care for key populations is fraught with difficulties (e.g., stigma)

One facilitator was concerned with how stigma could reduce care accessibility for key populations in Senegal, such as men who have sex with men and female sex workers, wondering about their safety if placed in IPT groups with participants from a non-stigmatized population.

*“Senegalese society is very resistant to such things, especially homosexuality, so we cannot include these people in group therapy with other people of heterosexual orientation. […] But we need to find a formula, because these people really need therapy.* [IPT-Facilitator 2].

##### 3.4.4.3 Transportation costs

Some IPT-participants and all IPT-facilitators identified transportation costs—to attend weekly group sessions—as a major treatment barrier. Most patients would use public transportation or take a cab to the treatment facility. For those with financial limitations, this presented a challenge.

*“It was sometimes difficult to come every Monday during therapy, because sometimes you want to come but do not have the transport [money]. But I did everything I could to come despite the difficulties.”* [IPT-Participant 5].

*“Implementation difficulties may be financial […]. Funds are needed to at least cover [patient] travel expenses, because most of the PLWH are in a very precarious financial situation.”* [IPT-Facilitator 2].

#### 3.4.5 Time burden for IPT-participants

Some challenges associated with group IPT were of a personal nature, such as schedule conflicts or competing interests (e.g., prayer and meal times; work).

“*My only problem was the timetable, as I came from far away and had to leave early sometimes without having breakfast. We’d get together [for group] at 2 pm on an empty stomach, thinking about the hours of prayer I’d have to catch up on since I did not get home until 6 pm.”* [IPT-Participant 6].

#### 3.4.6 Limited cultural applicability to the Senegalese context of some case examples in the IPT manual

IPT-facilitators also indicated that some examples given in the current version of the WHO manual (used in the implementation phase of group IPT) did not align well with Senegalese culture in terms of daily living context (i.e., socio-ecological realities).

*“In the IPT manual, the cases given as examples are cases that rarely occur in groups here.”* [IPT-Facilitator 2]

## 4 Discussion

The present qualitative study explored perceptions and experiences with group IPT among both IPT-participants and IPT-facilitators. Overall, the treatment was well perceived and received.

Though a number of IPT-participants had initially expressed some reluctance to be in group, they eventually overcame their hesitation and gradually built trust with other people in their respective groups. Concerns with HIV status getting “exposed” and social stigma regarding mental illness had shaped first impressions of group IPT. Confidentiality rules and psychoeducation around depression, on the other hand, seem to have alleviated IPT-participants’ fears and concerns. There was a growing ease with treatment going forward and group members sharing their interpersonal issues. IPT-participants spoke, listened, supported one another, and problem-solved in group. Following treatment, many stated they would recommend group IPT to others. It helped them recover, not just clinically but also interpersonally, and consequently many aspects of their life improved (e.g., work).

Group interventions are considered optimal in SSA, given their effectiveness and suitability for socialization, social support, networking, and interpersonal skill building (23, 24, 26, 36–38). Groups are also empowering; they promote acceptance and companionship (24, 36). The International Society for Interpersonal Psychotherapy asserts that IPT increases social functioning and support (39) which, in turn, has positive effects at the interpersonal and vocational levels. In the present study, IPT-participants reported that group IPT benefitted them in relationship and work. This may be a logical outcome of skill building in IPT, especially interpersonal skills. IPT-participants also learned useful problem-solving techniques, such as decision analysis, where one would identify options and decide on a viable course of action. Thus, group members have become better equipped to work through their difficulties as they actively participated in group and engaged in their recovery process.

Group IPT goes beyond the treatment of depression to its prevention inasmuch as it prepares participants to recognize early signs of relapse and proactively preempt its progress. This preparation includes concrete steps (e.g., seeking professional help, tapping one’s social support) and a set of proven techniques (e.g., the ones used to overcome the recent depression). Thus, group IPT may be said to protect patients against future depressive episodes. Further, it empowers them to become more self-reliant and practice self-care while concurrently building their social support base and expanding their skill repertoire through interpersonal engagement. Another observation is that group IPT helps to correct some misconceptions about depression among both IPT-participants and IPT-facilitators. In a society where mental disorders are stigmatized (40), this experiential reversal of what depression is and how it can be treated may be a first step in the fight against stigma and toward hope instillation. A second step, which may be taken concurrently, is to raise awareness (e.g., community) of depression as a treatable condition and the importance of its treatment.

With group IPT, PLWH came to perceive their HIV condition as a treatable and manageable illness. This can be seen as a logical extension to their group experience with depression, following IPT’s central premise of depression as a “treatable condition.” In terms of behavior, group members reported more commitment and adherence to HIV treatment—an important accomplishment in the context of the WHO 3×95 objectives (41). In this respect, the findings of the present study exceed those of a South African study in which PLWH receiving group IPT just recognized the importance of taking their HIV medication (24). Group IPT also appears to have helped participants overcome the negativity associated with HIV status and treatment. For instance, it positively transformed patient attitudes toward themselves and medical care.

IPT-facilitators also gained from group IPT. They expanded their helping skills (with group IPT competencies) and successfully assimilated the intervention into their professional milieu and practice. They reported that with group IPT they felt better equipped to help and support their patients. A study conducted in West Africa identified the lack of staff training as a main barrier to “task-shifting” and found it to be associated with poor understanding of depression (42). In learning and implementing group IPT with PLWH, the IPT-facilitators developed a broader understanding of mental illness, especially depression, and corrected the faulty notions they previously held about HIV. Group IPT also helped them grow professionally. They learned and successfully implemented an evidence-based intervention that proved effective with their population of interest (22); they expanded their counseling repertoire by learning general and IPT-specific group facilitation skills. Such a professional growth (e.g., skills) is likely to help support PLWH across the HIV care continuum. Further, such a skill building, and eventual capacity building in group IPT, could benefit the Senegalese healthcare sector beyond HIV treatment and care.

Building capacity with non-specialists still warrants some consideration. One notable challenge in tasking non-specialists with the treatment of psychiatric disorders is that it exposes them to difficulties they are not trained to deal with, such as emotional overload and secondary trauma. In the present study, IPT-facilitators reported that listening to some of their patients’ stories was emotionally taxing and at times difficult to bear. Therefore, additional training geared towards management of difficult emotions and secondary trauma seems warranted.

In terms of how group IPT was perceived upon implementation, a number of challenges were identified. For IPT-participants, the main concerns were confidentiality and trust (at the outset of treatment), in addition to accessibility issues (e.g., transportation cost; stigma). For IPT-facilitators, “time demands” were most important.

Confidentiality and privacy of information (e.g., HIV status) shared in group is a basic issue often reported in qualitative studies with PLWH (26, 36). As such, reluctance to join or speak in group about private matters is rather expected. In the present study, confidentiality rules and exceptions were explained to participants at the outset of therapy and repeated whenever necessary. Despite some initial hesitation, IPT-participants came to accept and value group sessions. They eventually came to see the group as a safe place and progressed in therapy.

However, successful, an intervention is useless when patients that it could potentially help cannot be found. In the present study, PLWH with depression were difficult to identify. The difficulties were mainly related to stigma, but also partly associated with procedure. Both could be resolved. The stigma challenge may be tackled with awareness raising campaigns. By targeting the mental health implications of living with HIV, for instance, one is likely to sensitize PLWH to the medical reality of their condition and open a help-seeking pathway (with appropriate counseling and referral). Of this population, a portion is likely to present for help with their depression. Other PLWH with depression may be identified directly by trained staff when accessing the healthcare system. In this vein, training social workers to identify depressed patients [e.g., mhGAP WHO manual (12)] would be valuable. Inasmuch as implementation of systematic depression screening is also necessary to facilitate access to care (43), we are currently evaluating a framework for integrating and systematizing this procedure as a healthcare routine.

Another major barrier to service access (and therefore treatment implementation) was transportation cost, especially for those patients living far from the treatment facility. This finding confirms what we pointed out in our quantitative and cost analysis studies (25, 44). Transportation issues are quite common in Africa. To illustrate, a study in South Africa identified healthcare site location as a major problem (in terms of distance to services), with recommendations to facilitate patient access to transportation and reduce travel fatigue (45). To be sure, accessibility remains a central issue in any program evaluation. Travel costs are an important barrier that can also drive up group IPT implementation costs, which calls for practical ways to improve treatment accessibility. One solution is to train personnel in more healthcare sites throughout the country so that group IPT becomes more accessible to patients living outside the capital, far from currently centralized service locations.

Treatment access, however, is not only a function of proximity. It is rather compounded by sociocultural factors. For example, stigma can compel patients to avoid facilities close to home for fear of being labeled and discriminated against in their community. In this vein, key populations (e.g., men who have sex with men) have more difficulty accessing care due to social, legal and stigma barriers—particularly in West Africa—and thus are exposed to a higher risk of mental health disorders, including depression (46, 47). As populations of interest (e.g., PLWH) remain highly stigmatized, finding solutions to improve treatment accessibility is critical. In this vein, interviewing study participants who had dropped out of therapy would have enabled us to gain a better understanding of such barriers.

Time demands due to group IPT activities and administration (e.g., recurring meetings; debriefings) were the main challenge reported by IPT-facilitators. This challenge, however, could be tackled within a task-shifting paradigm in line with a collaborative service delivery model. In the model, group facilitators (non-specialists) treating PLWH are helped by other facility staff, including specialists (48). This approach has two advantages: (1) it redistributes the burden of care in a more balanced way; and (2) it empowers group IPT facilitators and increases their resistance to burnout, especially when provided with a safe and supportive space for them to talk about their work and needs.

IPT-facilitators also pointed out that the WHO manual (used in training) could have benefited from minor revisions to enhance its cultural relevance. This was specific to some case illustrations deemed incongruent with Senegalese ways of life, since these illustrations in the WHO training manual were primarily based on field work in East Africa (e.g., Uganda). Notwithstanding, the issue could be easily resolved with some content adaptation (e.g., inclusion of cases from Senegalese life and culture). In effect, the WHO manual is clear that adaptation to local lifestyles, beliefs, and values is an integral part of its use and, therefore, its case illustrations cannot be taken literally across cultures and ecologies. However, if the point of content adaptation is raised here, it is primarily to alert researchers working cross-culturally to mind every aspect of their presentation, including examples used to illustrate IPT strategies and techniques—the prime targets of training. Related issues of cultural adaptation and remedies were described and discussed elsewhere (49).

Lastly, the above findings and recommendations pertain to the specific context of the study and its setting. In effect, it was conducted at a nationally-recognized university hospital, where working conditions were rather optimal compared to other healthcare facilities in the country. Future research should include other health care centers, preferably outside the capital (e.g., suburban/rural) to evaluate IPT perceptions, experiences and barriers in these different and less-centralized contexts. Participants, especially facilitators, may have emphasized positive experiences with group IPT because of social desirability towards the interviewer. However, we are confident that this prevarication bias was likely limited since the interviewer was independent of the treatment team and because qualitative data corroborated the quantitave results.

## 5 Conclusion

The present qualitative evaluation of group IPT among PLWH in Dakar, Senegal, gives an overall impression of intervention success. Study participants, be the IPT-participants or IPT-facilitators, reported positive experiences with group IPT. The description, elucidation, and subsequent discussion of these experiences provide a layer of understanding that cannot be attained by quantitative means alone. Participants were pleased with group IPT, both in terms of outcome as well as quality of experience. PLWH benefited clinically from group IPT, learned interpersonal skills and strategies to better manage their depression, and reported an improvement in quality of life that extended to the social and occupational domains. Group facilitators were able to learn and apply group IPT with relative ease and to integrate it into their work routine at the hospital, reporting increased self-efficacy, higher competence, and professional satisfaction. At a systems level, the use of a group intervention with a task-shifting approach appears to have been successful. As such, group IPT coupled with a task-shifting paradigm holds a promise in closing the mental health treatment gap.

The present study also highlights challenges that need to be addressed to facilitate group IPT implementation across the country. Since the study was conducted at a single facility (i.e., the national hospital in Dakar), additional studies are needed to evaluate facilitators and barriers to IPT implementation in other contexts. In this vein, future studies need to include different Senegalese facilities—with different systemic features and in various geographic locations—to assess how group IPT is perceived and experienced by PLWH and providers in variable healthcare settings and contexts.

## Data availability statement

The datasets presented in this article are not readily available because they contain confidential information that could compromise participant privacy but can be made available with non-identifiable aspects from the corresponding author upon reasonable request. Requests to access the datasets should be directed to charlotte.bernard@u-bordeaux.fr.

## Ethics statement

The study involving humans was approved by Conseil National d’Ethique de la Recherche en Santé (CNERS) in Dakar Senegal. The study was conducted in accordance with the local legislation and institutional requirements. The participants provided their written informed consent to participate in this study.

## Author contributions

CB: Conceptualization, Formal analysis, Methodology, Project administration, Writing – original draft, Data curation, Funding acquisition. IM: Formal analysis, Methodology, Writing – review & editing, Data curation. SZ: Supervision, Writing – review & editing, Methodology. JT: Investigation, Project administration, Writing – review & editing, Data curation. AD: Investigation, Project administration, Writing – review & editing, Data curation. NB: Methodology, Writing – review & editing, Supervision. IN: Data curation, Writing – review & editing, Supervision. OS: Writing – review & editing, Data curation, Supervision. HF: Conceptualization, Writing – review & editing. TB: Supervision, Writing – review & editing. LJ: Supervision, Writing – review & editing. HV: Writing – review & editing, Supervision. NN: Supervision, Writing – review & editing, Project administration. FD: Writing – review & editing, Funding acquisition, Conceptualization. MS: Supervision, Writing – review & editing, Project administration. NR: Conceptualization, Writing – review & editing, Funding acquisition, Methodology.

## Footnotes

^1^ https://www.who.int/fr/publications-detail/WHO-MSD-MER-16.4

^2^ https://www.iedea.org/regions/west-africa/
